# Prevalence of elevated cardiac troponin T in ICU patients using the high-sensitivity assay and the relationship with mortality

**DOI:** 10.1186/cc13383

**Published:** 2014-03-17

**Authors:** S O'Sullivan, M Sutton, G Fitzpatrick

**Affiliations:** 1 Tallaght Hospital & Trinity College Dublin, Ireland; 2 Health Research Board, Dublin, Ireland

## Introduction

Elevated cardiac troponin levels are common in ICU patients even in the absence of acute coronary syndromes and may be predictive of mortality. The recently introduced high-sensitivity cardiac troponin T (HS cTnT) assay has resulted in an increased detection of elevated cTnT in ICU patients [1]. The aim of this study was to determine the prevalence of elevated cTnT using the HS assay and its relationship with mortality.

## Methods

A retrospective observational study was performed on all ICU admissions over a 12-month period. Data were obtained from the clinical information system (ICIP; Philips) and the ICU audit databases (AcuBase). Data collected included patient demographics, peak cTnT value, APACHE II score, requirement for organ support and mortality. The primary outcome measure was hospital mortality. Data were analysed using SPSS v.17.0. cTnT levels were divided into categories for analysis: normal (<14 ng/l) and elevated. The elevated category was further subdivided into quartiles. Univariate analysis was performed between potential risk factors and mortality followed by multivariate regression analysis to ascertain independent predictors of mortality.

## Results

There were 417 admissions to the ICU during the study period, 89 of whom were excluded because of an absent cTnT value, leaving 328 patients included in the analysis. cTnT was elevated in 85% of patients. ICU mortality was 19% and hospital mortality was 28%. Hospital mortality (%) per cTnT category was: <14 ng/l = 2%; 14 to 38 ng/l = 19%; 39 to 90 ng/l = 26%; 91 to 252 ng/l = 39%; >252 ng/l = 43%. On univariate analysis, cTnT levels, age, ventilation and APACHE II score were significantly associated with mortality. cTnT levels were significant in multivariate regression independent of age and ventilation but did not reach significance (*P *= 0.06) in a multivariate analysis that included the APACHE II score.

## Conclusion

In 85% of general ICU patients, troponin measured by HS cTnT assay was elevated. cTnT levels were significantly associated with mortality and are predictive of mortality independent of age and mechanical ventilation, but not independently of APACHE II score. There was a high correlation between troponin levels and APACHE II scores. **Reference**
